# Animal Welfare Consequences of Organic Boar Fattening and Occurrence of Boar Taint on Five Commercial Farms

**DOI:** 10.3390/ani11102929

**Published:** 2021-10-10

**Authors:** Jeannette C. Lange, Anita Lange, Ute Knierim

**Affiliations:** 1 Farm Animal Behaviour and Husbandry Section, Faculty of Organic Agricultural Sciences, University of Kassel, Nordbahnhoftsr. 1a, 37213 Witzenhausen, Germany; uknierim@uni-kassel.de; 2 Department of Animal Sciences, Livestock Systems, Georg-August-University, Albrecht-Thaer-Weg 3, 37075 Göttingen, Germany; anita.lange@agr.uni-goettingen.de

**Keywords:** castration, agonistic behaviour, skin lesions, penis lesions, lameness, enriched housing

## Abstract

**Simple Summary:**

The usual castration of male fattening pigs is under discussion, especially in organic farming, because of the negative effects on the pig’s welfare and integrity. Nevertheless, it is feared that boars show more aggressive and mounting behaviours, resulting in stress, painful injuries and lameness, and that their meat has an unpleasant odour/taste (called “boar taint”). Therefore, it was examined to which extent these concerns come true under commercial organic conditions. Additionally, influences from management and husbandry were investigated, in order to identify conditions in organic fattening systems with low risk to welfare and meat quality. Despite more agonistic and mounting behaviours in boars compared to castrates, especially if housed next to females, no increased numbers of skin lesions, lame pigs, disease treatments or deaths were observed. Less skin lesions occurred when more space was provided. A moderate rate of wounds on penises was found, but not on farms with bulky straw bedding. A small number of carcasses (1.44%) were excluded from processing because of boar taint. In conclusion, boar fattening under the examined organic conditions appears to be a feasible alternative to castration, but penile injuries should be monitored at slaughter.

**Abstract:**

The welfare of male fattening pigs may be improved by refraining from castration, but may be compromised, in turn, by harmful social behaviour in groups of boars. In addition, boar taint may be problematic. This study aimed to evaluate these potential problems in boar fattening under commercial organic conditions. In total, 625 boars were compared with 433 barrows and 83 gilts regarding their social behaviour, lesions and lameness at 80 kg, before and after split marketing. The mixed-model analysis showed that significantly more short agonistic interactions, fights and mounting behaviours were observed in groups of boars. Agonistic interactions were reduced in spring/summer and when boars grew older. Fights and mounts were increased when boars had contact to female pigs in the neighbouring pen. No effect of split marketing, growth rate, homogeneity of groups, group size, feeding space and illumination hours could be detected. Increased interaction frequencies did not result in significantly more skin lesions, lameness, treatments or mortality. Increased space allowance reduced skin lesions. On 9.8% of the dissected boars’ penises, wounds were detected; they were absent on two farms with generous litter provision. Boar taint prevalence, as detected by human nose method, was 1.44%. Under the studied organic husbandry conditions, boar fattening appears to be practicable, although penile injuries should be monitored at slaughter.

## 1. Introduction

The fattening of entire boars represents one of the alternatives to the criticised practice of surgical castration without anaesthesia and analgesia, which inflicts pain on a great number of male pigs every year [1]. In the EU, there is some agreement that this procedure shall be discontinued [2]. In some EU countries, the fattening of entire boars has long been common practice (Ireland, United Kingdom, Portugal, Spain). In some it was introduced in the past years in a small proportion of the industry (e.g., Netherlands, Belgium, France, Germany), but mostly, male pigs are still castrated, either under anaesthesia, in line with national legal requirements (e.g., Norway, Sweden, Denmark, Switzerland, Germany) and EU requirements for organic farming, or without anaesthesia, in conventional farming (e.g., Eastern European countries) [3]. The use of entire boars entails a higher risk of tainted meat [4,5], and they may also be more aggressive, with adverse welfare effects due to injuries or social stress [6,7,8,9]. The extent of unwanted behaviours or resultant injuries, though, is influenced by various factors. The reported risk factors relate to large groups [7,10], changes in group composition from farrowing to slaughter [9], higher growth rates [7], homogeneous weight in the groups [11], certain genetics [5,12], scarce feeding space, insufficient water supply, suboptimal climate, too much noise (>80 db), low levels of certain amino acids in the feed, more prevalent illnesses in the pigs, higher fear of humans, as well as animal management lacking rest and routine [5]. Backus et al. [5] concluded that (the sum of) suboptimal conditions that are perceived as stressors constitute an increased risk for agonistic behaviour. Variable results were achieved regarding the effects of the contact with female pigs, with no effects on biting and fighting [13,14], less agonistic [8] or mounting behaviour [13] or more mounting [8,15]. Regarding split marketing, increased [16,17] or unchanged [13,18] agonistic interactions were found.

Organic pig husbandry differs in many aspects from common conventional farming. According to the EU regulation for organic pig husbandry, minimum requirements comprise, among others, at least 2.3 square metres per fattening pig (of 85–110 kg life weight (LW)) including an outdoor run and a lying area with bedding; roughage must be provided and up to half of the flooring may be slatted [19,20]. These conditions may alleviate some behavioural problems compared to conventional farming conditions. At the same time, the fattening of entire males agrees well with the organic principles in that not only the pain of castration is avoided, but the animal’s integrity is also preserved and no routine medication of anaesthetics, analgetics and antibiotics is needed [21]. On the other hand, because of the less intensive feeding regimes, organic pigs usually need more time to reach a certain slaughter weight [22,23]. An onset of puberty before slaughter may contribute to a higher aggressiveness. Stress reducing factors, e.g., access to roughage, may additionally contribute to lower rates of tainted meat, particularly to lower levels of skatole [24,25], whereas higher slaughter weights [26,27] or slaughter ages [5] may increase the risk of tainting. The onset of puberty and sex hormone production may be influenced by the contact with females. Higher [26] as well as lower [8] testicle weights in boars housed beside females were measured. Although the testicle weights at slaughter were not found to be related to the rates of tainted meat [8,26,28], they may reflect a sexual development in younger boars, which may influence the taint rates at slaughter age [29]. Further influencing factors such as the access to an outdoor run may enhance the increasing effects of the decreasing day length on the adrostenone levels [30], and the use of litter may result in more soiled pigs on warm days. Hygienic pen conditions and/or soiled pigs were found to be linked to higher skatole contents in fat tissues [5,31,32], but other investigations on soiling in relation to tainted meat did not confirm this [25,33,34].

The welfare consequences of the fattening of organic entire boars under organic [35,36,37] or near organic [14,38,39] conditions have already been investigated in a limited number of studies. Only the studies of Thomsen et al. [35,36] were carried out on commercial farms. The large variation in results, between the farms observed by Thomsen et al. [35,36] and between studies performed under conventional, organic or near organic conditions in general, reflects the abundance of potentially influencing factors and calls for further field studies. The aim of this study was therefore to evaluate boar fattening under commercial organic conditions in terms of potential welfare problems, namely to explore potential influencing factors on social interactions and their possible adverse consequences. Besides, the occurrence of tainted meat under these conditions was also included in the overall evaluation.

## 2. Materials and Methods

### 2.1. Experimental Design, Farms and Animals

Over 2.5 years, five commercial organic farms in North Rhine-Westphalia, Germany, participated in the study. In four to nine consecutive fattening cycles per farm, one or two groups of boars and one concurrent control group with barrows and sometimes gilts were monitored from birth to slaughter (at a live weight of around 120 to 130 kg). Male siblings of comparable weight were assigned to the boar and control groups, resulting in 43 groups of boars and 26 groups of barrows. Individual groups originated from 2 to 9 litters. Due to a lack of males, female siblings were added to reach the intended group size in 4 control groups; another 3 control groups exclusively consisted of female siblings. In total, 625 boars, 433 barrows and 83 gilts were included. All animals were crossbreds of Pietrain (Pi) sires and either purebred German Large White (DE) dams or dam lines of the breeding companies Topigs, JSR, Hypor and Hülsenberger (genetically based on Large White × Landrace crosses).

The weaned castrated and uncastrated piglets were raised together in larger groups at specialised farrowing farms, or in one case at the farrow-to-finish farm. Before and after the transfer to the finishing farms (at around 30 kg LW), the groups were split, but formerly unknown pigs never joined a group. The pigs were weighed when moving to the fattening unit and when the first pigs of that batch reached slaughter age (at 181 ± 36 days). The average daily weight gain and the homogeneity (variation coefficients of weight) per group were calculated on basis of the second weighing. Boar groups were either placed beside pens with barrows or male pigs only (25 groups) or beside pens with female pigs (gilts), which they could contact through the dividing bars (18 groups). Besides the pens with the monitored groups, each farm had a further 6 to 10 pens with gilts and barrows. For the distribution of gender, genetic lines and female contact per farm, see the Supplementary Material (Table S1).

The pigs were fed three times a day. The housing conditions complied with the EU regulations on organic farming [19,20] and are described in Table 1.

**Figure 1:**
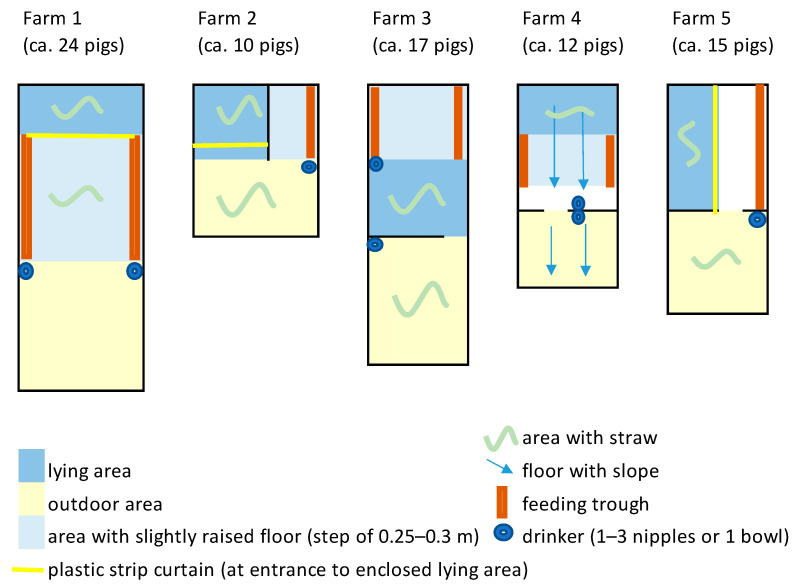
Pen design at the 5 farms.

All pens had solid (concrete) floors, covered with straw beddings of varying quantity (Table 1), and were structuredinto lying, feeding, activity and defecation areas in different ways (Figure 1). The outdoor runs also had solid floors and were roofed in varying proportions. The boar and control groups were housed in similar pens, and their assignment was reversed between fattening cycles.

The information on pen size, trough length, feeding system, drinkers and straw supply was recorded once per farm. The actual group sizes and illumination hours (daylight and artificial lighting) were recorded at the three times when behavioural observations were carried out. Air quality, particularly the ammonia content, was measured ongroup level when the slaughter age was reached, at pig snout’s height, with a Dräger Accuro^®^ gas detection pump (Ammonia 2/a^®^ tubes, mean of one measurement in the lying area and one in the activity area). The ambient temperature in the lying areas and the outdoor runs was measured half-hourly at the days of observation with a Voltcraft DL-111K data logger.

During transport and at the abattoir, the boars were kept together with their pen-mates and not mixed with unknown pigs, except in two cases where mixing with other slaughter pigs of the same farm occurred. The transport and waiting time were recorded for each delivery.

### 2.2. Behavioural Observations

Video recordings covering the whole pen were produced during 48 h at three times during fattening: when the average live-weight within a group was around 80 kg (“T1”), when first boars or age-matched barrows or gilts reached approximately 120 kg, i.e., the intended live-weight for slaughter (“T2a”), and within the first 48 h after the marketing of the first pigs of a group (“T2b”). While T1- and T2a-observations were always simultaneous in boars and controls, some T2b-observations had a different timing because of different weight gains in the groups. The equipment used were IR cameras Sony CCD 540 TVL (detec, Witzenhausen, Germany) and VTC-E 220/RP, 550 TVL (Santec BW AG, Ahrensburg, Germany), customary PCs, 32 bit, Windows XP/Windows 7, with a video grabber card RTV 24 (ADLINK, Mannheim, Germany) and the software 2_3_0 (Oliver Sanders, FLI, Celle, Germany). The behaviours were defined (Table 2) based on the definitions of Baumgartner et al. [40]. Their frequencies were determined with the Observer^®^ XT software (version 10, Noldus Information Technology, Wageningen, the Netherlands) and continuous behaviour sampling [41] by five trained observers after a sufficient inter-observer agreement, at r ≥ 0.70, had been reached (Table 2).

Behaviours within one metre around the trough were registered separately from those within the rest of the pen. The different behaviours observed within 15 min were highly correlated with those observed in 30 (*n* = 47 one-hour slots) and 60 (*n* = 3 one-hour slots) minutes/hour (Pearson’s correlation coefficient, r = 0.92–1). Therefore, the first 15 min per hour were observed. In 23% of the videos, each hour of the two consecutive recording days was included, but due to limitations in the observation time, this was limited in the remaining videos to every second hour, considering even hours on the first day and odd hours on the second day. The results from the hourly and 2-hourly observations were highly correlated (r = 0.96–0.99, *n* = 13, two-day slots). Periods of insufficient illumination (nights, ≤2 LUX) were excluded from the observations. If needed, this scheme was adapted, so that exactly two feeding events in every 48-h period were included. Due to technical problems, videos of three groups (2× boars and 1× barrows) could not be analysed. In eight further videos, that were shorter than 48 h (at minimum 24 h), missing hours were replaced by the corresponding hours (=odd hours) of the first day of observation. Always the same times were analysed for boars and controls that were kept in parallel. Altogether, 78 observations of 41 boar groups and 61 observations of 32 control groups, approximately equally distributed across T1, T2a and T2b, were analysed (Supplementary Material, Table S2). Ultimately, the applied scheme resulted in an effective observation time of 6.25 h per group, on average, ranging from a minimum of 2 h (1 observation) to a maximum of 13 h (3 observations) per group.

### 2.3. Scoring of Skin Lesions, Lameness and Dirtiness

All pigs were scored by one of two assessors regarding skin lesions (Table 3), lameness and dirtiness (Table 4) directly before or after the video recording (T1, T2a, T2b). For distribution of scorings and observations across farms, time points and seasons see Supplementary Material, Tables S2–S5. Both assessors were trained together, and the inter-observer reliability was tested before the on-farm scoring started (Table 3 and Table 4). On one side of the pigs’ body, the regions “ears”, “front”, “middle”, and “hind-quarters” (following Welfare Quality^®^, 2009) were inspected for lesions, with special attention paid to the penile region. Because soiling or pigmentation did not allow for a reliable scoring, 25 pigs were excluded from the assessment.

All lesions were summed unweighted for severity. Additionally, the lesions were weighted (medium wounds = 5 lesions, large wounds = 16 lesions) and the pigs were scored (0 = uninjured, 1 = moderately injured, or 2 = severely injured) according to the number of lesions per region following the Welfare Quality^®^ protocol [42].

### 2.4. Penis Dissections and Recording of Other Welfare Indicators

Altogether, 123 penises of boars from 16 slaughter batches (at least two per farm) and 14 penises of barrows from 4 batches were collected and dissected. Following Isernhagen [43], the lesion numbers were counted in four categories (0, 1–3, 4–7, 7–10 lesions), and regarding the kind of lesions (fresh, scars, both) and possible alterations of the crest of the penis (no, thickened, eroded). Furthermore, the incidences of disease treatments with antibiotics or antiphlogistics per group and fattening period as well as mortality (including reasons) were recorded by interviewing the farmers.

### 2.5. Boar Taint

All 625 carcasses of boars were checked at one of the two involved slaughterhouses for sensory deviations by official veterinarians, which had been trained to detect boar taint. Additionally, fat tissues of 544 of these boars were double-checked by a trained sensory panel (microwave-heated samples, panel and training: Food Technology Lab, University of Applied Sciences, Ostwestfalen-Lippe, Germany). The sensory panel agreed with all the taint classifications of the veterinarians. Boars with a strong unpleasant odour were sorted out, and their number was documented.

### 2.6. Data Processing and Statistics

The social interactions per pig and hour were calculated by multiplying the interactions by 4 and dividing by the number of observed pigs and by the number of observed hours, except for the interactions during the two feeding times, which were multiplied by 3 in the hourly observations and by 1.5 in the 2-hourly observations and divided by the number of observed pigs and hours instead, assuming a daily feeding time of approximately 45 min, from which 30 min of feeding time had been observed. The mean indoor temperature at the days of observation (6:30 a.m. to 6:30 p.m.) was classified into comfortable (>15 °C, including 4 cases of >25 °C which had no conspicuous effect on interactions), less comfortable (10–15 °C), uncomfortable (<10 °C) and very uncomfortable (<5 °C). Statistical analyses were conducted in R [44], version 3.5.3, and 4.1.1 for the agonistic interactions; descriptive statistics were conducted with the package pastecs, version 1.3–18 [45]. The possible effects on the frequencies of the repeatedly measured (T1, T2a, T2b) social interactions, the proportion of agonistic interactions around the feed trough in relation to the total agonistic interactions and the skin lesions were analysed on group level with linear mixed models of lme4, version 1.1 [46]. Random factors were “group”-nested within “batch” (=simultaneously raised groups of boars and controls) and within “farm”, except for the agonistic interactions where farm was used as a fixed factor (which were not significant), and group was only nested in batch to avoid an overfitted model. The fixed factor “status of castration/gender” was contained in every model. Furthermore, the factors “age”, “time of observation” (T1, T2a, T2b), “group size”, “space allowance”, “trough length per pig”, “season (metereological spring and summer versus autumn and winter)”, “illumination hours”, “contact with females”, “daily gain of weight per group” and “homogeneity of weight within group at slaughter age” were considered. The models were fitted by eliminating all fixed factors (except the factor “status of castration/gender”) from the saturated model that could not improve the model significantly (*p* < 0.05); in case of correlating factors (r ≥ 0.8), the less improving factor was dropped (comparisons of models with variance analysis). In the resulting models, furthermore, the possible interactions between the status of castration/gender and all the fixed factors that reached *p* < 0.1 in the modelling process were examined, regarding skin lesions, also between the group size and space allowance. The normality of distribution of the residuals was assessed graphically in histograms and quantil-quantil-plots with a 95% confidence interval. The homogeneity of variances was also assessed graphically from the plots of the distribution of residuals. In order to reach a normal distribution, agonistic interactions, fights and mounting were transformed to the power of 0.5, and skin lesions were logarithmised. However, the descriptive statistics are presented with untransformed data. The potential differences between boars and controls regarding lameness and mortality were analysed by a Chi-square-test.

## 3. Results

The environmental conditions and performance levels of the boars and controls during the study are presented in Table 5.

### 3.1. Social Interactions

**Figure 2:**
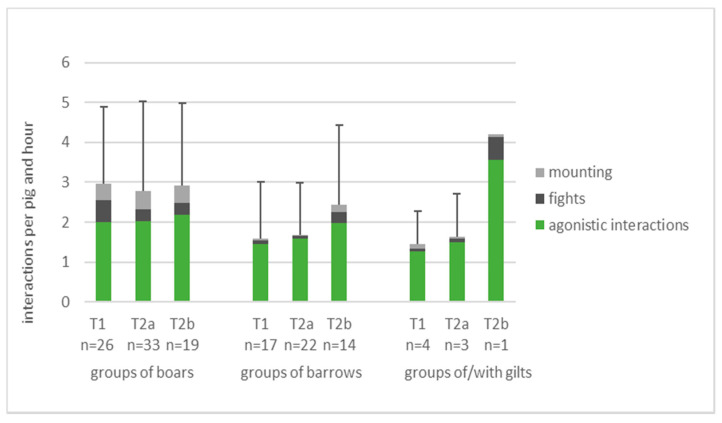
Social interactions per pig and hour in 41 groups of boars, 26 groups of barrows and 6 groups of gilts only or with gilts and barrows at three observation points (T1: mean pig weight around 80 kg, T2a: first pigs with ca. 120 kg, T2b: within 48 h after first split-marketing; *n* = number of observed groups per observation point).

The boars showed significantly more social interactions than controls (Table 6). Agonistic interactions lasting less than 5 s were most frequent (mean ± standard deviation over all observation times: boars: 2.1 ± 1.3, controls: 1.5 ± 0.9/pig × hour), followed by mounting behaviour (0.4 ± 0.5 versus 0.1 ± 0.1/pig*hour) and fighting (0.4 ± 0.4 versus 0.1 ± 0.2/pig × hour). Figure 2 shows the group means and standard deviation of interactions per observation point and separately for groups with only barrows and for groups of gilts only or of gilts and barrows.

**Figure 3:**
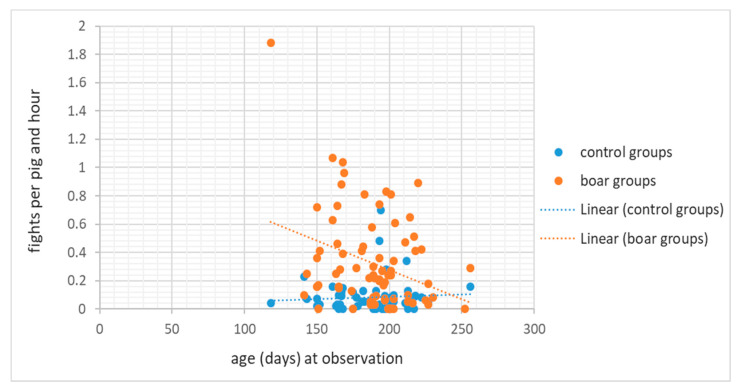
Significant interaction for frequency of fighting regarding the status of castration (boars vs. controls) and age.

Fights as well as mounting increased when the boars were kept beside female instead of male pigs (0.22 ± 0.31 versus 0.53 ± 0.28 fights/boar*hour, 0.28 ± 0.30 versus 0.56 ± 0.47 mounts/boar × hour; Table 6). Agonistic interactions and fights decreased with age or weight (time of observation), with a marked decline of fights in boars, but not in controls (interaction between age and castration status; Figure 3).

In boars, a smaller proportion of agonistic interactions (and fights) took place at the feeding trough than in the control groups (15% vs. 24%; Table 6). Seasonal influences were observed, with more agonistic interactions and fighting when the days were short (Table 6).

### 3.2. Skin Lesions

**Figure 4:**
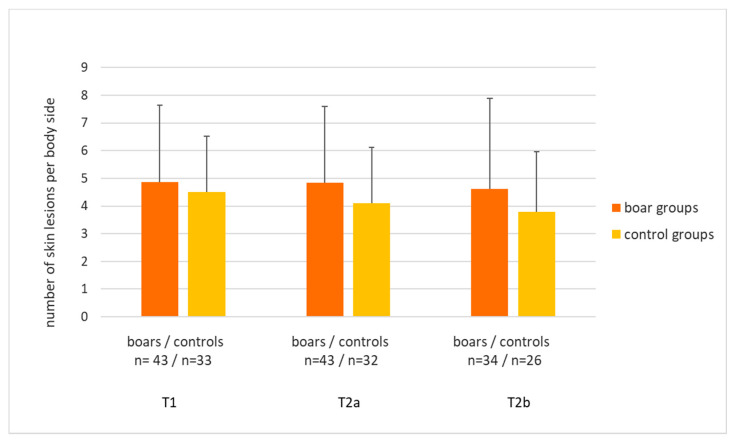
Average numbers of skin lesions on one body side in groups of boars (*n* = 43) and of barrows or gilts (*n* = 33) at the three points in time ((T1: mean pig weight around 80 kg; T2a: first pigs with ca. 120 kg; T2b: within 48 h after first split-marketing).

The numbers of skin lesions per body side did not significantly differ between boars and controls, although boars tended to have more lesions, but with a low effect size (Table 6, Figure 4, mean ± standard deviation: boars: 4.8 ± 3.0 lesions, *n* = 43 groups, controls: 4.1 ± 2.1 in, *n* = 33 groups). More space per pig resulted in significantly less skin lesions. The average numbers of lesions per pen ranged from 0.8 to 15.0 in boars and from 0.3 to 10.6 in controls. Ninety-eight per cent of the lesions were scratches or small wounds. Medium-sized wounds (boars: 0.08 ± 0.2; controls: 0.08 ± 0.1) and large wounds (boars and controls: 0.01 ± 0.1) were rare in boars and barrows or gilts alike. None of the large wounds was fresh and bleeding.

Classified according to Welfare Quality^®^ [42], 82.7% of boars compared to 86.9% of control pigs were uninjured, 17.1% vs. 13% moderately injured and 0.2% vs. 0.1% severely injured (there was an imprecision in the case of boars because of rounding). The resulting score for wounds on the body was 71.0 for the boars and 77.5 for controls, which would both be assessed as enhanced with some potential for improvement (acceptable: 40–60, enhanced: 60–80, excellent: 80–100, calculation see Welfare Quality^®^ [42], p. 71).

### 3.3. Penile Injuries, Lameness, Mortality, Disease Treatments

In the penile region, scratches were found only in single cases. One boar was found with fresh blood on the preputial sac. Penile lesions occurred on 12 of the 123 dissected boar penises (9.8%, Table 7) and on none of the 14 dissected barrow penises.

Farms were affected differently, with an average prevalence of injured penises in boars of 7.4%. All lesions were small (0.1 to 0.5 × 0.5 cm), except in one case, with 0.5 × 2 cm. One eroded crest and seven haematomas were found, four on penises with wounds and three on otherwise uninjured penises.

The prevalence of lameness (moderate and severe), did not significantly differ, but tended to be higher in controls (boars: T1: 0.2%, T2a: 0.2%, T2b: 1.1%; controls: T1: 0.8%, T2a: 0.6%, T2b: 1.7%, χ^2^_(1, n=2725)_ = 3.78, *p* = 0.05). This was also the case when additionally mild cases of lameness were included in the category of lame pigs (boars: 2.1% versus controls: 3.2%, χ^2^_(1, n=2725)_= 3.11, *p* = 0.08).

The mortality did not significantly differ between boars (1.8%) and controls (1.7%; χ^2^_(1, n=2725)_ = 0.02, *p* = 0.9). The cases of disease treatments during fattening were exceptional and well below 1% in boars and controls alike.

### 3.4. Boar Taint

At the slaughter line, 1.44% of boars were excluded from further processing because of boar taint. This number of tainted boars was too low for statistical analysis, while the descriptive data (Table 8) show no conspicuous differences between affected and unaffected boars.

## 4. Discussion

### 4.1. Social Interactions

Nearly 50% more agonistic interactions, including fights, were observed in boars compared to controls (barrows and gilts). In agreement with other studies [15,47,48], but contrary to Thomsen et al. [35], this reflects a higher activity and possibly aggressiveness in boars compared to castrates. However, the effect of the season on agonistic interactions (without fighting) was as strong as that of the castration status. The total average of 2.5 agonistic interactions and fights per hour and boar is comparable to the results from other studies under enriched or organic housing conditions, with 1.7 to approx. 4.7 interactions per hour and boar [14,35,36]. The wide range of agonistic interaction levels within and between studies in general, from 1.7 [35] to 19.0 [49] interactions per hour and boar versus 1.0 [14] to 10.8 [16] in barrows or gilts, suggests that other influences may be even more important, although methodical differences between studies will also play a role. In the current investigation, the competition for feed seemed to be a minor reason for conflicts in boar groups, as the proportion of agonistic interactions and fights taking place around the feeding trough was higher in the control groups. In contrast to earlier investigations [8,13,14], we found that housing female pigs in the neighbouring pens increased the fighting in the boar groups. Except for female contact, season, and age, no further associations between observed behaviours and management or housing factors could be detected, possibly due to the enriched and spacious housing conditions on all five organic farms. Moreover, the air ammonia contents of more than 20 ppm, for instance, that might negatively affect social behaviour [50] were reached only in two measurements. 

The seasonal effect consisted of more agonistic interactions in autumn and winter than in spring and summer. This was not linked to illumination hours (including artificial lighting), as they were included in the model but were never significant. Prunier et al. [51] found more skin lesions in autumn. Like us, they did not observe more mounting. Furthermore, they did not detect higher androstenone levels in the fat during that time. Wild boars, as short day breeders, are more sexually active, with elevated sex hormone levels, in October to February [24], but this rhythm in androstenone levels is only rudimentarily preserved in domesticated boars [29]. Moreover, we observed seasonally increased agonistic interactions in boars and barrows alike, but no seasonal effects on mounting behaviour. One hypothetical mechanism could be the increased competition for feed, triggered by a higher need to store energy because of the imminent winter. However, this should be further studied. 

Agonistic interactions and fighting declined with age and/or weight. Other authors also observed a decline in agonistic behaviours as pigs got older ([36]: at 3, 4 and 5 months of age) or increased in weight ([35]: at 40, 70 and 90 kg; 46: at 67 and 109 kg; [14]: between 30 and 120 kg). Thomsen et al. [35] pointed at possible relationships with decreasing activity levels with age, confirmed by Isernhagen [43], who found a high correlation between overall activity and fights. Thus, the expectation of increasing aggressiveness in boars with increasing sexual maturation was not fulfilled. The decline in fighting with age was significantly stronger in boars than in controls. Furthermore, fights but not agonistic interactions were less frequent without than with female contact, which may promote some kind of display behaviour. On the other hand, part of the behaviour classified as fights during our observations may even have been play behaviour. This could not be clearly differentiated based on the applied definition. In future work, more attention should be paid to a better distinction, possibly with the additional help of acoustic data. In addition, increased fighting in young pigs might be an expression of social exploration that promotes knowledge about relative fighting abilities and social competence [52]. Further reported influencing factors, such as homogenous groups displaying more bites and longer fights [11] during the mixing of piglets at 7 weeks of age could not be confirmed in the present investigation (without mixing) regarding agonistic and mounting behaviour, which was in line with investigations of castrated and female weaners [53,54,55].

Moreover, mounting was largely increased in boars (nine times as much as in controls). This in conformity with earlier studies, where frequencies ranged from 0.015 to 16 mounts per boar and hour [43] and 0.001 to 2.5 per barrow or gilt and hour, influenced by the age at observation, the durations of the observations and environmental factors. Our results are near the lower end of the range. In contrast to Salmon and Edwards [8], we observed at least doubled numbers of mounting (as well as fights) when boars had contact with females. In line with Thomsen et al. [35], but unlike others [14,16,56], we did not find decreasing frequencies with age. Isernhagen [43] observed even more mounting towards the slaughter age in boars, but not in barrows. The different findings may point to different motivations of mounting which are difficult to distinguish. Potentially playful mounting should decrease during the fattening period, while sexually motivated mounting should increase in parallel to the levels of androstenone [51]. Dominance-related mounting should be associated with other agonistic interactions, e.g., due to competition for resources. Hintze et al. [56] regarded general arousal and “fence mounting” as further causes of mounting. In our case, mounting was not conspicuously concentrated at the “fences”. 

### 4.2. Consequences of Social Interactions

The more frequent social interactions in boars did not lead to significant increases of skin lesion numbers, lameness prevalences, disease treatment incidences and mortality rates, although a tendency to more lesions in boars was found. Penis lesions in low numbers could be detected in boars, but not in barrows. 

The level of skin lesions in the present study (approximately 9 per pig) was in the middle range compared to other studies using similar scoring schemes ([17]: 6.7 per boar at slaughter age; [57]: 8–10.5 per barrow or female pig, depending on the group size and space allowance; [51]: approx. 13 per boar, late fattening in spring, and 32 per boar, early fattening in autumn). In addition, only very few severe injuries were recorded both in boars and controls. The discrepancy between distinctly higher numbers of agonistic interactions and fights in boars than in barrows, but about similar numbers of injuries, suggests that fights may often have been playful or explorative, rather than aggressive. In five boar groups and one barrow group, however, conspicuously higher average numbers of lesions, mostly coupled with a greater variance, suggest that in some cases agonistic interactions may escalate and result in more injuries in boars than in barrows. A careful observation of boars, especially in autumn and winter, may allow to intervene in such cases. 

The relatively spacious and enriched conditions on the five farms will have been conducive to avoiding bites after threats or to showing submission in the course of agonistic interactions which depend on the available space and pen structures [58,59]. Although space allowances on the farms exceeded the minimum requirements [19], with 2.3 to 5.2 m^2^pig^−1^ before the first pigs were marketed and 2.8 to 14 m^2^pig^−1^ thereafter, increased space was still associated with less lesions. 

Regarding the consequences of mounting, our results agree with those of Salmon and Edwards [8] and Hintze et al. [56], who also found no increased skin lesions despite more frequent mounting in boars. Nevertheless, we noticed at the slaughter line that, sometimes, haematomas of distinct shape became visible after scalding, dehairing and washing, which pointed to mounting as a cause. Their systematic recording would be worthwhile in the future. Additionally, sometimes high-pitched vocalisations indicated distress in the mounted pig. Hintze et al. [56] differentiated presumably sexual mounting with pelvic thrusts and/or protruded penis from otherwise motivated mounting. They observed sexually motivated mounting almost exclusively in boars (approx. 50% of all mountings). It lasted longer, and sometimes provoked high-pitched vocalisations (approx. 15% of all mountings). 

In line with other studies [15,35,56], but in contrast to Rydhmer et al. [7], we did not observe more boars being lame than controls. Differing results may depend on the amount of available straw bedding with cushioning properties. In fact, one of the participating farms with restrictive straw provision contributed over-proportionately to the identified cases of lameness. 

Further potential consequences of mounting with protruded penis are injuries of the penis due to biting by pen mates, probably as an exploratory behaviour. Isernhagen [43] reported conventional fattening systems in which more than 80% of dissected penises of boars were injured. Often, several wounds, fresh and older ones, were found side by side and were likely due to bites from pen-mates. In contrast, Holinger et al. [38] reported 2.8% injured penises under enriched conditions. In our study, an intermediate proportion of 9.8% of dissected boar penises were injured, mostly with just one lesion. Only once was fresh blood on a boar’s preputial sac found, either from being bitten or from a superficial erosion of the mucosal membranes caused by the contact with another pig’s back. Injured penises were not evenly distributed across farms; on one farm 9 out of 35 dissected penises were injured, while on two farms no injuries were found in the 28 dissected penises. A contributing factor might have been the sparse straw provision in the lying area and no straw in the activity area on the farm with higher prevalence, compared to the bulky straw bedding in the activity and lying areas of the other two farms. Based on the 14 dissected penises of barrows, which consistently showed adherences between the penis and its serous membranes (frenulum praeputii), we can confirm that barrows obviously never protrude their penis during mounting. The frenulum praeputii persists in early castrated males because the macerating effect of androstenone is lacking [42,60]. 

### 4.3. Research Design

This on-farm study was carried out to test whether the results from controlled small-scale experiments can be transferred to commercial farm settings where the different combinations of multiple housing and management factors may lead to deviating effects. On-farm studies, on the other hand, have to cope with commercial limitations. For instance, the piglet producers were not able to deliver enough male piglets to assign only barrows to the control groups. As we found no significant differences between females and barrows or mixed groups (Supplementary Material, Table S6), the inclusion of female siblings appears acceptable. Furthermore, the multitude of housing and management factors presents a challenge. By monitoring the aspects for which effects on social behaviour have been reported, and by including them in the statistical modelling, we wanted to exclude potential bias, but we were also interested in finding husbandry factors with strong effects regardless of individual farms. However, except for space allowance concerning skin lesions, no particular risk or preventive factor could be identified. Possible reasons besides the enriched conditions on all farms mentioned above are an overriding of potential effects by the random farm effect. Moreover, some conditions, like very low or very high temperatures or high ammonia concentrations, occurred so infrequently that we refrained from including them in the statistical analysis. Therefore, the non-significant results do not provide any proof that these factors do not matter, but under the investigated conditions their effect was either low, due to interactions with other factors, or the variation was not high enough within farms or in the whole sample.

### 4.4. Prevalence of Tainted Carcasses

An unpleasant odour was detected in 1.44% of the slaughtered boars. Backus et al. [5] found on average 3.31% of 1.7 million slaughtered boars from 1.583 farms in the Netherlands to be tainted. Thus, the prevalence obtained in our study was low, although compared to conventional fattening, the boars were slaughtered at higher ages. However, this prevented further analysis. The descriptive data provide no indication of any effect of potential influencing factors on tainting, like weight, age, husbandry conditions, contact with females or split marketing, although potential genetic influences should be further investigated. The “human nose” method for the detection of tainted boars, which was used in this study, may not correlate well with chemically analysed boar taint compounds [61]. Therefore, the validity of this method may be questioned. However, if the assessors are well trained, this method is deemed to be robust enough [62] for classifications at the slaughter line. Furthermore, all carcasses assessed as untainted had been marketed without receiving any complaint.

## 5. Conclusions

Significantly more agonistic interactions and mountings in boars than in barrows or gilts did not lead to significantly more skin lesions, cases of lameness or disease treatments or mortality under the organic conditions investigated. This indicates that these housing and management conditions allow for a more active boar behaviour without a considerably increased risk of welfare problems. However, penile injuries in boars were found in moderate quantity; they were absent on two farms with generous litter provision. The percentage of tainted carcasses was low. We conclude that under the studied organic husbandry conditions and from an animal welfare perspective, boar fattening presents a practicable alternative to castration, although a monitoring of penile injuries should be carried out at slaughter.

## Figures and Tables

**Table 1:** Selected housing and management conditions of the five investigated farms.

Farm No.	Group Size ^a^ Median (Min–Max)	Space Allowance ^a^ (m^2^pig^−1^)	Feeding Space (m^2^pig^−1^)	Feeding System	Drinker	Straw Supply ^b^ (Bedding Depth)
1	24 (20–26)	2.4–3.1	0.38–0.50	Liquid-feed, long-trough	nipple	moderate
2	10 (6–11)	2.8–5.2	0.36–0.67	Dry feed, long trough	bowl	ample
3	17 (14–20)	2.3–3.3	0.30–0.43	Wet-dry feed ^c^, long trough	nipple with trough	ample
4	12 (9–12)	2.3–3.1	0.33–0.44	Dry feed, long trough	nipple	sparse
5	15 (13–17)	2.3–3.0	0.29–0.38	Dry feed, long trough ^d^	nipple	moderate

^a^ Given are the initial group sizes and space allowances per group, that were further reduced during the fattening cycle by sporadic losses and split marketing; ^b^ depth of bedding typical for the farm (ample: ≥20 cm in the lying and activity areas; moderate: 5–20 cm in the lying area and ≤5 cm in the activity area; sparse: ≤5 cm in the lying and activity areas); ^c^ porridge-like feed: dry feed mixed with water in the feeding trough directly before consumption; ^d^ the boar group of the 4th fattening cycle was fed with a wet-dry feeder.

**Table 2:** Definitions of social interactions] and results of inter-observer reliability testing (Pearson correlation coefficient) between observers 1–3 (I) and observers 2–5 (II) using 12 video sequences of 15 min duration each.

Behaviour	Definition	Inter-Observer Agreement (r)		
			Mean	Min.–Max.
Agonistic interactions	Biting, head knocking, or attempt, with displacement of the receiver, duration < 5 s	I:	0.87	0.86–0.89
		II:	0.84	0.76–0.96
Fighting	Agonistic interaction, optionally interspersed by chasing, spinning round each other or parallel pressing, duration ≥ 5 s	I:	0.92	0.88–0.95
		II:	0.94	0.89–0.98
Mounting	Front legs on both sides of the lying or standing receiver	I:	0.95	0.91–0.97
		II:	0.95	0.93–0.97

**Table 3:** Definitions of the scores regarding skin lesions (modified from Welfare Quality^®^, 2009) and results of inter-assessor reliability testing between two assessors (Pearson correlation coefficient r and proportion of matchings; *n* = 182 pigs).

Measure/Score	Definition	Number of Animals	Count (Mean of 2 Assessors)	Correlation Coefficient; Proportion of Matchings
Skin lesions	Unweighted sum of scratches and wounds in all states of healing, from fresh and bleeding to just a detectable crust	182	411	r = 0.99; 86%
Scratches	Linear surface penetration of the epidermis (width ≤ 0.5 cm, at ears ≤0.3 cm), length ≥ 2 cm		384	r = 0.99; 90%
Wounds:	Surface penetration involving tissue beneath the epidermis (width > 0.5 cm, at ears >0.3 cm)			
- small	length < 2 cm		19	r = 0.70; 98%
- medium	length 2–5 cm		8	r = 0.82; 98%
- large	length > 5 cm		0	-; 100%

**Table 4:** Definitions of the scores regarding lameness and dirtiness (modified from Welfare Quality^®^, 2009) and results of the inter-assessor reliability testing between two assessors (Prevalence-adjusted bias-adjusted kappa: PABAK; *n* = 182 pigs and 11 videos for lameness).

Measure/Score	Definition	Number of Animals	Proportion (Mean of 2 Assessors)	PABAK ^a^
Lameness		182 ^b^/11 ^c^		0.99 ^b^/0.76 ^c^
0	regular gait, all four limbs evenly loaded		97% ^b^/32% ^c^	
1	difficulties in walking, swagger of caudal body while walking, shortened stride		2% ^b/^27% ^c^	
2	severe lameness, bearing minimal weight on a limb, visible while walking and while standing		1% ^b^/23% ^c^	
3	bearing no weight on a limb, or unable to walk		0% ^b^/18% ^c^	
Dirtiness		182		0.84
0	≤20% of body-surface soiled		60%	
1	>20 ≤50% of body-surface soiled		23%	
2	>50% of body-surface soiled		17%	

^a^ prevalence-adjusted bias-adjusted kappa; used because of the categorical data with uneven distribution of cases across the categories; ^b^ live scoring; ^c^ scoring from videos because of the low numbers of lame pigs and missing score 3-cases.

**Table 5:** Environmental conditions at the days of observation and body weight measures.

Factor	Measure	Boars	Controls
Illumination hours *	hours of natural or artificial lighting indoor and outdoor per day	11.5 (min: 6, max: 16)	
Indoor temperature	mean indoor temperature (6:30 a.m.–6:30 p.m. at observed 2 days)	>15 °C: 80%, 10–15 °C: 15%, <10 °C: 4%, <5 °C: 1%	
Air ammonia content	mean of lying and activity areas	5.1 ppm (0% > 10 ppm, 3% > 20 ppm)	
		Boars	Controls
		mean (min–max)	
Weight homogeneity *	variation coefficient (SD/mean) of body weights per group when first pigs per batch at ca. 120 kg	0.13 (0.07–0.22)	0.13 (0.05–0.39)
Growth rate *	group mean of daily weight gain, calculated from weights when first pigs of a batch at ca. 120 kg minus 1.5 kg (approximate birth weight) divided by days from birth to weighing (on average 183 days)	540 g (421–689)	566 g (414–734)

* included in modeling, but did not stay in the final models.

**Table 6:** Final results of linear mixed models regarding possible effects on different social interactions.

Behaviour	Factor	Model Estimate	T-Value	*p*-Value
Agonistic interactions (duration of <5 s)	Status of castration (boars versus controls)	0.20	4.31	<0.001
	Age (days)	−0.003	−2.74	0.007
	Season (1.9–28.2 vs. 1.3–31.8)	0.196	2.62	0.01
Fighting (agonistic interactions ≥5 s)	Status of castration (boar versus control)	1.18	4.74	<0.001
	Contact with females (boars with versus without contact)	0.29	5.13	<0.001
	Season (1.9–28.2 vs. 1.3–31.8)	0.11	2.63	0.01
	Age (days)	−0.0002	−0.20	0.84
	Interaction of status of castration with age	−0.04	−2.95	0.004
Proportion of agonistic interactions and fights taking place at the trough	Status of castration (boars vs controls)	−0.18	−5.43	<0.001
Mounting	Status of castration (boars versus controls)	0.50	7.74	<0.001
	Contact with females (boars with versus without contact)	0.19	2.76	0.007
Skin lesions	Status of castration (boars vs controls)	0.09	1.90	0.06
	Space allowance (m^2^ per pig)	−0.06	−4.29	<0.001

**Table 7:** Absolute and relative number of boars with penis lesions and the kind of lesions, *n* = 123 boars (scoring scheme: Isernhagen, 2015).

Numbers of Dissected Penises		Number (Proportion) of Penises in the Different Lesion Classes					Kind of Lesions (Numbers of Penises)		
	n	0	1–3	4–6	7–10	>10	Fresh/crusted	scar	both
Farm 1	44	42 (95%)	2 (5%)	0	0	0	1	1	0
Farm 2	14	14 (100%)	0	0	0	0	0	0	0
Farm 3	14	14 (100%)	0	0	0	0	0	0	0
Farm 4	35	26 (74%)	9 (26%)	0	0	0	8	1	0
Farm 5	16	15 (94%)	1 (6%)	0	0	0	1	0	0

**Table 8:** Retrospective comparison of tainted and untainted boars concerning potentially influencing conditions around slaughter.

	Tainted Boars (*n* = 9)		Untainted Boars (*n* = 616)	
	Mean	Range	Mean	Range
Slaughter weight (kg)	93	75–120	92	46–126
Slaughter age (days)	229	173–261	216	145–297
Average daily gain (ADG) of weight (g)	525	451–630	544	421–689
Homogeneity of weights (SD/mean, at T2a ^a^)	0.14	0.09–0.27	0.12	0.05–0.19
Group size (the week before slaughter)	11	5–26	11	2–26
Air ammonia concentration	5.1	1–7	5.4	2–26
Affected pigs per genetic dam-line (sire-lines: Pietrain)				
DE (German Large White)	0.8%		99.2%	
JSR	0.5%		99.5%	
Topig	3.2%		96.8%	
Others ^b^	0.8%		99.2%	
Frequency of agonistic behaviour in the group (per pig × hour, at T2a)	1.8	0.6–3.0	2.4	0.2–5.4
Frequency of mounting in the group (per pig × hour, at T2a)	0.3	<0.1–1.0	0.4	0–2.0
Number of skin lesions (per body-side, at T2a)	5.0	0–13	5.0	0–34
Percentage of soiled pigs (>20% of body surface, at T2a)	11%		24%	
Duration of transport (hours)	0.75	0.25–2.5	0.74	0.25–2.5
Waiting time at slaughter (hours)	3.3	0–10	4.4	0–10

^a^: T2a: the first pigs of a group to reach ca. 120 kg. ^b^: mainly dams of the German breeding company “Hülsenberger Zuchtschweine”.

## Data Availability

The data presented in this study are available on request from the corresponding author.

## Supplementary Materials

The following are available online at https://www.mdpi.com/article/10.3390/ani11102929/s1, Table S1: Number of groups (boars, barrows, females and mixed gender) and pigs (in brackets) per farm, number of boar groups with and without contact to females in the neighbouring pen, and genetic line of pigs. Table S2: Numbers of groups and pigs (in brackets) per farm observed at the different time points T1, T2a, T2b*, Table S3: Numbers of groups and pigs (in brackets) scored regarding skin lesions and lameness at observation time points (T1, T2a, T2b*) per farm, Table S4: Seasonal distribution of scoring of groups of boars and controls (and number of pigs scored in the respective season*), Table S5: Seasonal distribution of observations of groups of boars and controls (and number of pigs observed in the respective season*), Table S6: Behaviour and skin lesions in groups of barrows, females, and those of mixed gender (estimate, T-value and p-value from mixed models; fixed factor: gender with four levels, boars, mixed, females and barrows; random factor: group within batch within farm); prevalence of lameness, disease, and death in barrows, female pigs, and boars.
