# Supplementary material for: Animal Welfare Consequences of Organic Boar Fattening and Occurrence of Boar Taint on Five Commercial Farms
Source: Animals (Basel). 2021 Oct 10;11(10):2929. doi: 10.3390/ani11102929 (PMC8532605; doi:10.3390/ani11102929)
Supplement: Supplementary file 1 [file animals-11-02929-s001.zip › animals-1377803-supplementary.pdf]

animals MDPI

Supplementary material – for the article: Animal Welfare Consequences of Organic Boar Fattening and Occurrence of Boar Taint on Five Commercial Farms

Table S1. Number of groups (boars, barrows, females and mixed gender) and pigs (in brackets) per farm, number of boar groups with and without contact to females in the neighbouring pen, and genetic line of pigs.

Farm	Groups (pigs)	Entire males			Barrows only	Females only	Barrows + females	Genetic line (dams)
			Contact to females					
			with	with-out				
1	16 (392)	8 (191)	3	5	7 (177)	--	1 (24)	Others/ Topig*
2	21 (199)	12 (120)	5	7	9 (79)	--	--	Topig
3	12 (207)	6 (100)	3	3	6 (107)	--	--	JSR
4	17 (198)	11 (128)	4	7	3 (35)	1 (12)	2 (23)	DE
5	10 (145)	6 (86)	3	3	1 (15)	2 (29)	1 (15)	JSR
1-5	76 (1.141)	43 (625)	18	25	26 (413)	3 (41)	4 (62)	

* genetics of the 8 batches (simultaneously raised groups of boars and controls) of farm 1: 6 of them were mixtures of different lines, based on Hypor and Hülsenberger breeding pigs, and 2 batches had Topig dams.

Table S2. Numbers of groups and pigs (in brackets) per farm observed at the different time points T1, T2a, T2b*.

	Boars			Control pigs		
Farm	T1	T2a	T2b	T1	T2a	T2b
1	3 (70)	4 (95)	4 (52)	3 (73)	4 (96)	4 (56)
2	9 (89)	10 (100)	3 (13)	7 (65)	8 (68)	3 (11)
3	4 (68)	6 (98)	4 (35)	4 (73)	6 (105)	4 (36)
4	8 (92)	9 (104)	4 (32)	5 (57)	5 (56)	2 (17)
5	2 (28)	4 (58)	4 (34)	2 (29)	2 (29)	2 (18)
1-5	26 (347)	33 (455)	19 (166)	21 (297)	25 (354)	15 (138)

* T1: around 80kg live weight, T2a: first pigs of a group with 120kg, T2b: 48 hours after marketing of the first pigs of a group.

Table S3. Numbers of groups and pigs (in brackets) scored regarding skin lesions and lameness at observation time points (T1, T2a, T2b*) per farm.

	Boars			Control pigs		
Farm	T1	T2a	T2b	T1	T2a	T2b
1	8 (190)	8 (187)	8 (96)	8 (194)	7 (169)	8 (118)
2	12 (120)	12 (120)	8 (42)	9 (79)	9 (78)	6 (29)
3	6 (98)	6 (98)	5 (46)	6 (105)	6 (105)	5 (47)
4	11 (128)	11 (128)	8 (60)	6 (69)	6 (67)	4 (29)
5	6 (86)	6 (86)	5 (40)	4 (58)	4 (58)	3 (28)
1-5	43 (622)	43 (619)	34 (284)	33 (505)	32 (477)	26 (251)

* T1: around 80kg live weight, T2a: first pigs of a group with 120kg, T2b: 48 hours after marketing of the first pigs of a group.

Table S4. Seasonal distribution of scoring of groups of boars and controls (and number of pigs scored in the respective season*).

	Spring (1.3.-30.5.)		Summer (1.6.-30.8.)		Autumn (1.9.-30.11.)		Winter (1.12.-28.2.)	
Farm	Boar groups	Control groups	Boar groups	Control groups	Boar groups	Control groups	Boar groups	Control groups
1	--	--	12 (240)	9 (185)	6 (118)	9 (183)	6 (115)	5 (103)
2	14 (126)	9 (74)	5 (46)	4 (28)	12 (103)	10 (74)	1 (7)	1 (10)
3	--	--	6 (83)	6 (83)	6 (76)	4 (63)	5 (83)	7 (111)
4	9 (91)	5 (56)	10 (115)	5 (53)	4 (35)	2 (10)	7 (75)	4 (46)
5	2 (31)	1 (14)	10 (120)	8 (109)	6 (75)	2 (21)	--	--
1-5	24 (248)	15 (144)	43 (604)	32 (468)	35 (407)	27 (351)	19 (280)	17 (270)

*: The (meteorological) season in which a group of pigs was scored was recorded. Due to the repeated measurements, the same group may occur in two different seasons.

Table S5. Seasonal distribution of observations of groups of boars and controls (and number of pigs observed in the respective season*).

	Spring (1.3.-30.5.)		Summer (1.6.-30.8.)		Autumn (1.9.-30.11.)		Winter (1.12.-28.2.)	
Farm	Boar groups	Control groups	Boar groups	Control groups	Boar groups	Control groups	Boar groups	Control groups
1	--	--	6 (124)	4 (104)	1 (24)	3 (56)	4 (69)	4 (78)
2	10 (100)	6 (56)	3 (25)	2 (12)	9 (77)	9 (66)	1 (7)	1 (10)
3	--	--	5 (68)	5 (83)	5 (61)	3 (46)	4 (72)	6 (100)
4	9 (91)	5 (56)	7 (79)	4 (42)	2 (24)	1 (8)	3 (34)	2 (24)
5	--	--	5 (59)	4 (55)	5 (61)	2 (21)	--	--
1-5	19 (191)	11 (112)	26 (355)	19 (296)	22 (247)	18 (197)	12 (182)	13 (212)

*: The (meteorological) season in which a group of pigs was observed was recorded. Due to the repeated measurements, the same group may occur in two different seasons.

To test whether barrows and females in control groups led to comparable results, mixed models for each of the behaviours and for skin lesions were calculated with group nested in batch nested in farm as random and gender (barrows, mixed, females, boars) as sole fixed factor. In these models, agonistic interactions were not transformed, fights and mounts were transformed to the power of 0.5, and skin lesions were logarithmized (Table S6).

Table S6. Behaviour and skin lesions in groups of barrows, females, and those of mixed gender (estimate, T-value and p-value from mixed models; fixed factor: gender with four levels, boars, mixed, females and barrows; random factor: group within batch within farm); prevalence of lameness, disease, and death in barrows, female pigs, and boars.

	Groups of females (n=3, versus groups of barrows, n=26)	Groups of mixed gender (n=3, versus groups of barrows)	Groups of boars (n=33, versus groups of barrows)

Agonistic interactions	-0.33, T=-0.76,p=0.45	0.08, T=0.0.23, p=0.82	0.45, T=3.44, p=0.002
Fights	-0.08, T=-0.56, p=0.58	0.09, T=0.77, p=0.44	0.27, T=6.17, p<0.001
Mounting	0.06, T=0.41, p=0.68	0.03, T=0.22, p=0.83	0.40, T=7.36, <0.001
Skin lesions	-0.004, T=-0.02, p=0.98	-0.23, T=-1.70, p=0.10	0.06, T=1.04, p=0.30
